# Improved adulticidal activity against *Aedes aegypti* (L.) and *Aedes albopictus* (Skuse) from synergy between *Cinnamomum* spp. essential oils

**DOI:** 10.1038/s41598-021-84159-z

**Published:** 2021-02-25

**Authors:** Jirapon Aungtikun, Mayura Soonwera

**Affiliations:** Department of Plant Production Technology, Faculty of Agricultural Technology, King Mongkut’s Institute of Technology Ladkrabang, Ladkrabang, Bangkok, 10520 Thailand

**Keywords:** Entomology, Natural products

## Abstract

Improved natural adulticidal agents against mosquito vectors are in urgent need, and essential oils from Cinnamomum plants can assume this role quite readily. *Cinnamomum verum*, *C*. *cassia*, and *C*. *loureiroi* essential oils (EOs) were extracted from the barks and evaluated for their chemical composition by GC–MS. The major constituent of the three EOs was cinnamaldehyde. WHO susceptibility tests on individual and combined EOs as well as cinnamaldehyde were conducted against female adults of *Aedes aegypti* and *Aedes albopictus.* All EO combinations exhibited a synergistic effect, manifesting a higher toxicity, with a synergistic value ranging from 2.9 to 6.7. Their increasing mortality value was improved between 16.0 to 41.7%. The highest synergistic effect was achieved by an EO combination of 0.5% *C*. *cassia* + 0.5% *C*. *loureiroi*, while the highest insecticidal activity was achieved by 2.5% *C*. *verum* + 2.5% *C*. *cassia* and 1% cinnamaldehyde, with a knockdown and mortality rate of 100% and a KT_50_ between 0.7 and 2.1 min. This combination was more toxic to both mosquito species than 1% w/v cypermethrin. These findings demonstrate that cinnamaldehyde and synergistic combinations of *C*. *verum* + *C*. *cassia* EOs and *C*. *cassia* + *C*. *loureiroi* EOs have a high insecticidal efficacy against *Aedes* populations.

## Introduction

*Aedes aegypti* (L.) and *Aedes albopictus* (Skuse) were widespread in many parts of Thailand^1^. Also known as dengue mosquito vectors, they play a predominant role in the transmission of dengue fever, dengue hemorrhagic fever, and other infectious viral diseases such as Zika, chikungunya and yellow fever^1,2^. Among these diseases, dengue hemorrhagic fever is the most severe viral disease caused by four dengue viral serotypes (DEN-1, 2, 3, and 4)^1^. These diseases spread quickly in many parts of the world. Annually, more than half of the world’s population, an estimated 3.9 billion people in more than 150 countries, are at risk of infection with dengue viruses. Many dengue cases (70%) have been reported in Southeast Asian countries such as the Philippines, Vietnam, Bangladesh, Malaysia, and Thailand^3–6^. In 1954, the first severe outbreak of dengue occurred in the Philippines. Four years later (1958), the first dengue outbreak occurred in Thailand^7^. Currently, the spread of dengue in Thailand is on an increasing trend^8^. The Ministry of Public Health of Thailand reported that the total dengue cases in the year 2017, 2018, and 2019 were 53,190; 85,849; and 121,696 cases, respectively, with 63, 111, and 144 deaths, respectively. The estimated dengue cases for 2020 was over 140,000^8^. Most importantly, there is no effective dengue vaccine against all four dengue viral serotypes, thus mosquito vector control was considered the best strategy for preventing the disease. There are several strategies for controlling and managing mosquito vectors^7,8^, but chemical control is the strategy that has been used worldwide and extensively in everyday life. A chemical control can act as a larvicide, an adulticide, or a repellent^7,8^.

Most chemical insecticides exert some serious negative effects on human health, the environment, pollinators (bee, bumble bee, carpenter bee, stringless bee etc.), parasitic and predatorial insects (braconids, trichogramma, and ichneumonids). To make matters even worse, rapid insect resistance to them has rendered most of them ineffective nowadays. Chemical resistance has been reported to occur in *Aedes aegypti* (*Ae*. *aegypti*) and *Aedes albopictus* (*Ae*. *albopictus*) populations worldwide^9–13^. In particular, *Ae*. *aegypti* and *Ae*. *albopictus* have been reported to be resistant to organochlorines (DDT), organophosphates (malathion), carbamate (carbaryl), and pyrethriods (permethrin, and deltamethrin)^14,15^.

Consequently, safe and high efficacy alternatives for mosquito vector control have been urgently searched for and developed. Plant extracts, especially plant essential oils (EOs), have shown dominant activity against mosquitoes and other insect pests. They are highly promising as safe alternatives to chemical insecticides^15–19^. EOs are safe for human health and the environment, as they have been declared to be low-risk active substances by European Food Safety Authority (EFSA)^20–22^. They do not pollute the environment but rapidly degrade in soil and water. Moreover, it is difficult for mosquito vectors to develop resistance towards them^23^. More than 122 EOs from 26 plant families have a mosquito control efficacy, such as those from *Alpinia galanga*, *Anethum graveolens*, *Amomum villosum*, *Amomum krervanh*, *Artemisia verlotiorum*, *Cannabis sativa*, *Cananga odorata*, *Carlina acaulis*, *Curcuma zedoaria*, *Cymbopogon citratus*, *Cymbopogon nadus*, *Eucalyptus globulus*, *Foeniculum vulgare*, *Illicium verum*, *Lavandula dentata*, *Pimpinella anisum*, *Ruta chalepensis*, *Zanthoxylum limonella*, *Zingiber cassumunar*, and *Zingiber mekongense*. They are toxic to the adults and larvae of *Ae*. *aegypti*, *Ae*. *albopictus*, *Anopheles dirus*, and *Culex quinquefasciatus*^24–35^. EOs have not only been used singly but also in combinations. Combinations of different EOs can be synergistic in their mosquito vector control efficacy^36,37^. Combinations of *Ocimum sanctum* + *Mentha piperita* EOs and *E*. *globulus* + *Plectranthus amboinicus* EOs showed synergistic repellency activity against *Ae*. *aegypti* females^38^. *C*. *citratus* + *E*. *globulus* EOs showed a synergistic insecticidal activity against *Ae*. *aegypti*, *Ae*. *albopictus*, and *Musca domestica* females^28^. EO combinations of *Syzygium aromaticum* + *I*. *verum*, *S*. *aromaticum* + *Trachyspermum ammi*, *I*. *verum* + *T*. *ammi*, *T*. *ammi* + *Pelargonium graveolens*, *Satureja montana* + *Aloysia citriodora*, and *S*. *montana* + *A*. *citriodora* showed synergistic larvicidal activities against *Ae*. *aegypti* and *Culex quinquefasciatus*^39,40^.

EOs from *Cinnamomum* spp. show several dominant activities for mosquito control: *C*. *verum* EO show repellency activity against *Ae*. *aegypti* and *Cx*. *quinquefasciatus* adults^41^. *C*. *verum*, *C.damhaensis*, *C. longipetiolatum*, *C. ovatum*, *C. polyadelphum*, and *C. tonkinense* EOs showed a strong larvicidal activity against *Ae*. *aegypti* and *Cx*. *quinquefasciatus* larvae^42,43^. *C*. *verum* EO also showed a strong adulticidal acidity against *Aedes aegypti* adults^44^. Many studies have reported the efficacy of *Cinnamomum* EOs against mosquitoes, but none has focused on the possible synergy in adulticidal activity between two combined EOs from *Cinnamomum* spp*.* The purpose of this study was to determine the adulticidal activities of individual *C*. *verum*, *C*. *cassia*, and *C*. *loureiroi* EOs, the activities of their major constituents, and the activities of several of their combinations against adult females of *Ae*. *aegypti* and *Ae*. *albopictus*. We had selected to investigate these three EOs among numerous plant EOs because they have been reported to possess pharmaceutical, antifungal, antibacterial and insecticidal properties as well as to be safe for human and the environment^45–48^. EO combinations that showed a highly synergistic effect can be developed into effective adulticidal agents for controlling and managing *Aedes* mosquitoes in urban and rural areas as well as for controlling dengue diseases and other vector-borne diseases^31,36^.

## Results

### Chemical compositions of the three *Cinnamomum* spp. EOs

**Figure 1:**
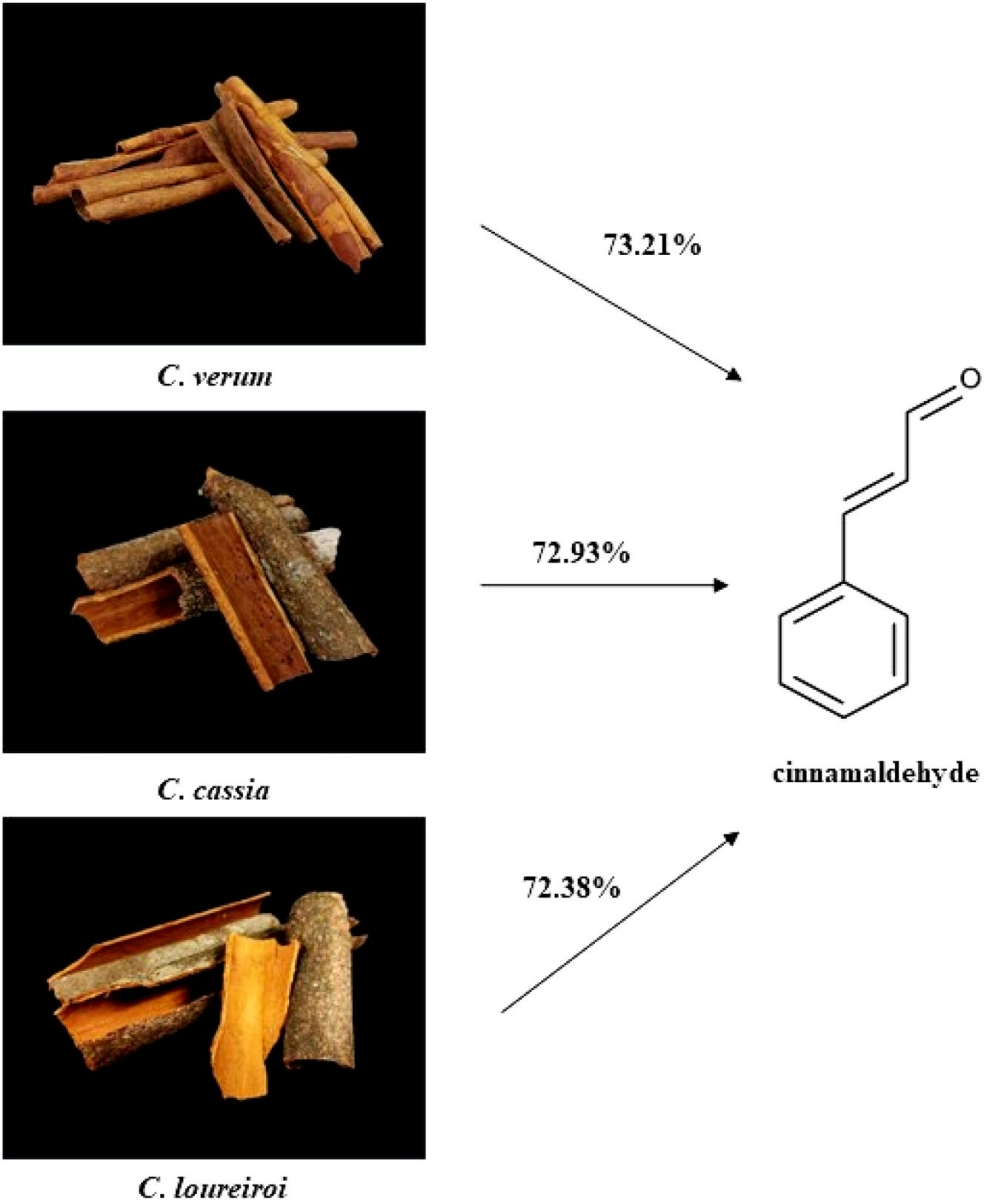
Barks of *C*. *verum*, *C*. *cassia*, and *C*. *loureiroi*; % cinnamaldehyde of three *Cinnamomum* spp. EOs and chemical structure of cinnamaldehyde.

Hydro-distillation of *C*. *verum*, *C*. *cassia*, and *C*. *loureiroi* barks provided pale yellow and pale tan EOs. The highest essential oil yield was obtained from *C*. *cassia* (1.12% v/w), followed by *C*. *verum* (1.01% v/w) and *C*. *loureiroi* (0.82% v/w). The chemical compositions of the three *Cinnamomum* spp. EOs were analyzed by GC–MS. A total of 15, 15, and 11 chemical constituents were identified from *C*. *verum*, *C*. *cassia*, and *C*. *loureiroi* EOs, respectively, accounting for 98.24, 98.60 and 97.07% of their composition, respectively, as presented in Table 1. Cinnamaldehyde was the major constituent of these three *Cinnamomum* spp. EOs. Its chemical structure is displayed in Fig. 1. The highest cinnamaldehyde content of 73.21% was found in *C*. *verum* EO; the second highest was 72.93% in *C*. cassia EO; and *C*. *loureiroi* EO had the lowest cinnamaldehyde content at 72.38% of its chemical composition.

**Table 1:** Physical property, chemical constituents of *C*. *verum*, *C*. *cassia*, and *C*. *loureiroi* essential oils.

No	Constituent	RI^a^	KI^b^	Percentage of total composition			IM^c^
				*C*. *verum*	*C*. *cassia*	*C*. *loureiroi*	
1	α-Pinene	933	933	0.84	0.87	–	MS,RI
2	Camphene	952	952	0.57	0.68	0.78	MS,RI
3	β-Myrcene	991	991	0.45	–	–	MS,RI
4	α-Phellandrene	1003	1003	0.41	0.38	–	MS,RI
5	Benzyl alcohol	1009	1009	12.83	–	–	MS,RI
6	Limonene	1033	1033	0.54	0.65	–	MS,RI
7	1,8-Cineole	1039	1039	0.57	0.22	–	MS,RI
8	Acetophenone	1075	1076	–	1.21	1.93	MS,RI
9	Linalool	1111	1111	–	1.21	1.49	MS,RI
10	Camphor	1117	1118	–	0.98	0.87	MS,RI
11	Benzenepropanal	1127	1128	–	3.47	0.97	MS,RI
12	Borneol	1170	1171	1.13	2.86	2.11	MS,RI
13	Cinnamaldehyde	1221	1222	73.21	72.93	72.38	MS,RI
14	Eugenol	1355	1355	1.29	–	–	MS,RI
15	Methyl cinnamate	1364	1364	0.28	–	–	MS,RI
16	Copaene	1381	1381	1.83	3.75	4.63	MS,RI
17	Cinnamyl acetate	1414	1414	2.51	3.13	5.42	MS,RI
18	β-Caryophyllene	1417	1418	–	0.64	–	MS,RI
19	Cedrene	1426	1427	–	0.84	0.79	MS,RI
20	α-Guaiene	1432	1433	–	–	4.86	MS,RI
21	Cinnamic acid	1462	1462	0.45	–	–	MS,RI
22	Cadalene	1657	1658	0.21	–	–	MS,RI
	Total identified (%)			98.24	98.60	97.07	
	Yield (% v/w)			1.01	1.12	0.82	
	Color			Pale yellow	Pale tan	Pale tan	

^a^RI = Retention index analyzed with HP-5 MS column, experimentally determined using standard alkanes (C_7_–C_30_).

^b^KI = Kovats index from https://pubchem.ncbi.nlm.nih.gov and NIST (https://webbook.nist.gov).

^c^IM = Identification methods; MS, mass spectrum matching with chemicals in the computer mass library of Adams^61^.

Minor constituents of *C*. *verum* EO were benzyl alcohol (12.83%), cinnamyl acetate (2.51%), copaene (1.83%), eugenol (1.29%), borneol (1.13%), α-pinene (0.84%), camphene (0.57%), 1,8-cineole (0.57%), limonene (0.54%), β-myrcene (0.45%), cinnamic acid (0.45%), α-phellandrene (0.41%), methyl cinnamate (0.28%), and cadalene (0.21%). Minor constituents of *C. cassia* EO were copaene (3.75%), benzenepropanal (3.47%), cinnamyl acetate (3.13%), borneol (2.86%), acetophenone (1.21%), linalool (1.21%), camphor (0.98%), α-pinene (0.87%), cedrene (0.84%), camphene (0.68%), limonene (0.65%), β-caryophyllene (0.64%), α-phellandrene (0.38%), and 1,8-cineole (0.22%). Finally, minor constituents of *C*. *loureiroi* EO were cinnamyl acetate (5.42%), α-guaiene (4.86%), copaene (4.63%), borneol (2.11%), acetophenone (1.93%), linalool (1.49%), benzenepropanal (0.97%), camphor (0.87%), cedrene (0.79%), and camphene (0.78%).

### Toxicity of the three EOs on *Ae*.* aegypti* and *Ae*.* albopictus* females and their synergistic effect

**Figure 2:**
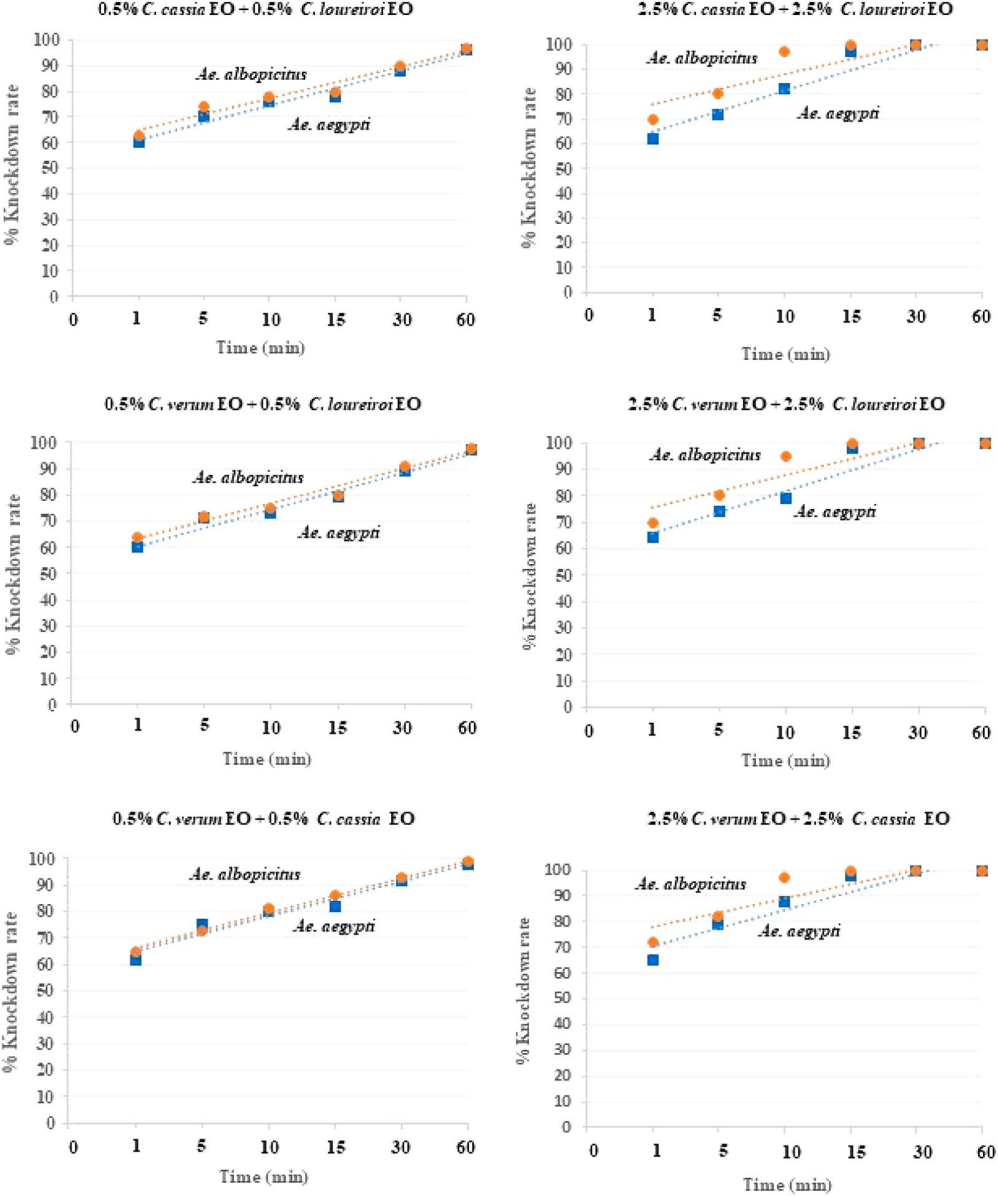
Linear regression between % knockdown rate and exposure time (min) of combinations of EOs against females of *Ae*. *aegypti* and *Ae*. *albopictus*.

The efficacies of individual EOs from *C*. *verum*, *C*. *cassia*, and *C*. *loureiroi* and several of their combinations against females of *Ae*. *aegypti* and *Ae*. *albopictus* were evaluated, at 60 min after treatment, in terms of knockdown rate (K), 50% knockdown time (KT_50_), increasing knockdown value (IKV), effective knockdown index (EKI) and synergistic value (SV), presented in Tables 2, 3 and Fig. 2. According to the obtained KT_50_ values, *Ae*. *albopictus* females were more susceptible to every tested EO and EO combination than *Ae*. *aegypti* females. Moreover, all EO combinations from *C*. *verum*, *C*. *cassia*, and *C*. *loureiroi* were more toxic to the females of both species than either the individual *C*. *verum*, *C*. *cassia*, or *C*. *loureiroi* EOs alone, with a knockdown rate ranging from 96.0 to 100%, a KT_50_ ranging from 2.1 to 3.2 min, a KT_90_ ranging from 8.2 to 32.4 min against *Ae*. *Aegypti,* as well as a KT_50_ ranging from 1.8 to 2.6 min and a KT_90_ ranging from 6.3 to 28.9 min against *Ae*. *albopictus*. The combination of 2.5% *C*. *verum* + 2.5% *C*. *cassia* EOs achieved the highest knockdown rate with a KT_50_ of 2.1 min and a KT_90_ of 8.2 min against *Ae*. *aegypti* and with a KT_50_ of 1.8 min and a KT_90_ of 6.3 min against *Ae*. *albopictus*. All EO combinations exhibited a synergistic effect, manifesting a higher toxicity than that of individual EOs, to both species, with an SV of 2.9–6.8. Their %IKV was improved by 7.4 to 19.3% compared to those of individual EOs. The highest synergistic effect against both species was achieved by 0.5% *C*. *cassia* + 0.5% *C*. *loureiroi* EOs with an SV of 5.5 to 6.6 and an IKV of 16.0–18.3%. Combinations of 2.5% *C*. *verum* + 2.5% *C*. *cassia* EOs, 2.5% *C*. *verum* + 2.5% *C*. *loureiroi* EOs, and 2.5% *C*. *cassia* + 2.5% *C*. *loureiroi* EOs exhibited a high synergistic effect. They were more toxic to both mosquito species than 1% w/v cypermethrin, with an effective knockdown index of 0.8 to 0.9. Every other treatment was less toxic against both species than 1% w/v cypermethrin. Not surprisingly, 70% v/v ethyl alcohol (negative control) did not cause any knockdown at all and was non-toxic to the females both mosquito species (0% knockdown rate).

**Table 2:** Knockdown rates and KT_50_ time of essential oils from *C*. *verum*, *C*. *cassia*, and *C*. *loureiroi* and their combinations against females of *Ae*. *aegypti*.

Treatment	Knockdown rate (%) ± SD/time (min)	KT_50_ (min) (LCL-UCL)	KT_90_ (min) (LCL-UCL)	Slope ± SE	R^2^	Chi-square (χ^2^)	IKV (%)	SV	Status	EKI
	60									
CV1	88.8 ± 2.1d	12.3 (8.3–16.2)	44.5 (37.4–55.9)	0.040 ± 0.003	0.555	143.722	–	–	–	4.40
CC1	78.4 ± 3.1e	17.6 (13.4–22.1)	61.5 (51.9–76.4)	0.029 ± 0.003	0.573	103.575	–	–	–	6.29
CL1	80.6 ± 2.6de	19.0 (14.8–23.6)	59.5 (50.4–73.9)	0.032 ± 0.003	0.534	115.841	–	–	–	6.79
CV5	92.6 ± 2.6b	7.7 (3.0–11.7)	35.1 (28.4–47.1)	0.047 ± 0.004	0.492	212.224	–	–	–	2.75
CC5	90.4 ± 2.3c	9.6 (5.6–13.2)	40.6 (33.9–51.3)	0.041 ± 0.004	0.600	142.482	–	–	–	3.43
CL5	88.8 ± 2.3d	15.0 (11.5–18.6)	46.5 (39.8–56.9)	0.041 ± 0.003	0.622	124.966	–	–	–	5.36
M1	98.4 ± 2.0ab	2.6 (0.5–7.1)	25.2 (18.0–44.7)	0.056 ± 0.006	0.600	426.017	9.8, 20.3	4.7, 6.8	Synergy	0.93
M2	97.6 ± 1.4ab	3.2 (0.6–7.1)	30.3 (23.8–42.6)	0.047 ± 0.005	0.571	198.581	9.0, 17.4	3.8, 5.9	Synergy	1.14
M3	96.0 ± 1.3ab	3.2 (1.1–6.9)	32.4 (25.8–44.6)	0.043 ± 0.004	0.587	175.353	18.3, 16.0	5.5, 5.9	Synergy	1.14
M4	100a	2.1 (1.6–3.6)	8.2 (7.9–11.3)	0.193 ± 0.017	0.692	118.463	7.4, 9.6	3.7, 4.6	Synergy	0.75
M5	100a	2.3 (2.1–4.1)	10.8 (9.2–13.0)	0.166 ± 0.015	0.583	103.139	7.4, 11.2	3.4, 6.5	Synergy	0.82
M6	100a	2.4 (2.2–4.2)	10.8 (8.2–32.4)	0.170 ± 0.015	0.677	97.225	9.6, 11.2	4.0, 6.3	Synergy	0.85
1% w/w cypermethrin	100a	2.8 (2.5–3.2)	11.1 (7.5–13.8)	0.197 ± 0.018	0.697	105.323	–	–		–
70% v/v ethyl alcohol	0f.	ns	ns	ns	Ns	ns	ns	ns		ns

Mean percentage knockdown rates in each column followed by a different letter are significantly different (one way ANOVA and Duncan’s multiple range test, *P* < 0.05).

*KT*_*50*_ 50% knockdown time, *R*^*2*^ regression coefficient, *LCL *lower confidence limit, *UCL* upper confidence limit, *IKV (%)* Increasing Knockdown Value, *EKI* Effective Knockdown Index, *SV* Synergistic Value, *ns* not significant. Treatment codes are defined in Table 1.

**Table 3:** Knockdown rates and KT_50_ time of essential oils from *C*. *verum*, *C*. *cassia* and *C*. *loureiroi* and their combinations against females of *Ae*. *albopictus*.

Treatment	Knockdown rate (%) ± SD/time (min)	KT_50_ (min) (LCL-UCL)	KT_90_ (min) (LCL-UCL)	Slope ± SE	R^2^	Chi-square (χ^2^)	IKV (%)	SV	Status	EKI
	60									
CV1	80.1 ± 2.8c	12.0 (6.4–17.0)	59.3 (48.5–72.0)	0.027 ± 0.003	0.461	126.775	–	–	–	6.0
CC1	80.2 ± 2.6c	14.5 (10.0–18.9)	59.6 (49.9–75.1)	0.028 ± 0.003	0.594	104.450	–	–	–	7.25
CL1	80.8 ± 1.7c	14.3 (9.4–19.2)	61.0 (50.5–78.6)	0.027 ± 0.003	0.509	115.768	–	–	–	7.15
CV5	92.0 ± 1.8b	5.5 (2.0–9.8)	34.1 (27.2–46.9)	0.045 ± 0.004	0.457	219.808	–	–	–	2.75
CC5	90.4 ± 2.3b	8.6 (4.4–12.3)	40.7 (33.9–51.6)	0.040 ± 0.004	0.618	137.977	–	–	–	4.30
CL5	90.4 ± 2.6b	6.7 (1.7–10.8)	38.0 (31.0–50.0)	0.041 ± 0.004	0.495	174.495	–	–	–	3.35
M1	99.2 ± 1.5a	2.2 (0.9–1.9)	21.6 (12.0–1149.4)	0.066 ± 0.007	0.560	1625.786	19.3, 19.2	5.5, 6.6	Synergy	1.1
M2	98.4 ± 2.1a	2.6 (1.1–6.9)	26.9 (20.4–41.2)	0.053 ± 0.006	0.676	273.714	18.6, 17.9	4.6, 5.5	Synergy	1.3
M3	97.6 ± 2.1a	2.2 (0.9–6.1)	28.9 (22.4–42.0)	0.047 ± 0.005	0.658	217.692	17.8, 17.2	6.6, 6.5	Synergy	1.1
M4	100a	1.8 (1.2–2.6)	6.3 (5.3–7.9)	0.292 ± 0.028	0.557	117.266	8.0, 9.6	3.1, 4.8	Synergy	0.9
M5	100a	1.9 (1.4–2.9)	7.0 (6.0–8.6)	0.262 ± 0.025	0.999	101.032	8.0, 9.6	2.9, 3.5	Synergy	0.95
M6	100a	1.9 (1.4–2.7)	6.5 (5.6–8.0)	0.288 ± 0.028	0.999	104.165	8.0, 9.6	4.5, 3.5	Synergy	0.95
1% w/w cypermethrin	100a	2.0 (1.5–2.8)	6.5 (5.5–8.2)	0.278 ± 0.026	0.999	105.255	–	–	–	–
70% v/v ethyl alcohol	0f.	ns	ns	ns	ns	ns	ns	ns		ns

Mean percentage knockdown rates in each column followed by a different letter are significantly different (one way ANOVA and Duncan’s multiple range test, *P* < 0.05).

*KT*_*50*_ 50% knockdown time, *R*^*2*^ regression coefficient, *LCL* lower confidence limit, *UCL* upper confidence limit, *IKV (%)* Increasing Knockdown Value, *EKI *Effective Knockdown Index, *SV *Synergistic Value, *ns* not significant. Treatment codes are defined in Table 1.

Mortality rates (M) at 24 h after exposure against the females of *Ae*. *aegypti* and *Ae*. *albopictus* of individual EOs—*C*. *verum*, *C*. *cassia*, and *C*. *loureiroi* EOs—and their combinations as well as those achieved by 1% w/v cypermethrin and 70% v/v ethyl alcohol are tabulated in Table 4. Regarding the outcomes of knockdown and mortality rate assays, for a treatment of an insecticidal agent, it is quite possible that the mosquitoes may be knocked down after a short period of time but may recover and did not die afterward, so the knockdown rate may be high but the mortality is low. This was not the case in this study: a higher knockdown rate also came with a higher mortality rate against both species (Tables 2, 3). Females of both species were more susceptible to the EO combinations (100% mortality) than the individual EOs (57.8–88.8% mortality). Individual *Cinnamomum* spp. EOs achieved a mortality rate of 69.6–88.8% against *Ae*. *aegypti* females and 57.8–84.0% against *Ae*. *albopictus* females. They were less toxic (EMI < 1) to both mosquito species than 1% w/v cypermethrin. Every combination with 0.5% *Cinnamomum* spp. EOs exhibited a mortality rate against *Ae*. *aegypti* females from 98.4 to 99.3%, an IMV of 21.1–29.9% and a mortality rate against *Ae*. *albopictus* females from 99.2 to 99.7% and an IMV of 27.4–41.7%. The highest IMV was achieved by the combination of 0.5% *C*. *cassia* + 0.5% *C*. *loureiroi* EOs. The IMV achieved by combinations of EOs was improved by 23.6–29.3% against *Ae*. *aegypti* females and by 27.4–41.7% against *Ae*. *albopictus*. Their adulticidal activities were higher than that of 1% w/v cypermethrin with an EMI < 1.0. The highest insecticidal activity was achieved by the combinations of 2.5% *C*. *verum* + 2.5% *C*. *cassia* EOs, 2.5% *C*. *verum* + 2.5% *C*. *loureiroi* EOs, and 2.5% *C*. *cassia* + 2.5% *C*. *loureiroi* EOs, with 100% mortality rates, a 11.2–19.2% improvement in IMV against *Ae*. *aegypti* as well as a 16.0–18.4% improvement in IMV against *Ae*. *albopictus*. Their adulticidal activities were equivalent to that of 1% w/v cypermethrin with an EMI of 1.

**Table 4:** Mortality rates (MR), increasing mortality value (IMV) and effective mortality index (EMI) of essential oils from *C*. *verum*, *C*. *cassia*, and *C*. *loureiroi* and their combinations against females of *Ae*. *aegypti* and *Ae*. *albopictus*.

Treatment	*Ae*. *aegypti*			*Ae*. *albopictus*		
	MR (%)	IMV (%)	EMI	MR (%)	IMV (%)	EMI
CV1	77.6 ± 3.1b	–	0.78	72.0 ± 4.2a	–	0.72
CC1	69.6 ± 4.3bc	–	0.70	58.4 ± 3.8c	–	0.58
CL1	75.2 ± 2.4b	–	0.75	57.8 ± 2.3c	–	0.58
CV5	83.2 ± 2.7ab	–	0.83	84.0 ± 4.9ab	–	0.84
CC5	80.8 ± 2.1ab	–	0.81	82.4 ± 2.1ab	–	0.82
CL5	88.8 ± 2.3ab	–	0.89	81.6 ± 3.3ab	–	0.82
M1	99.3 ± 1.5a	21.9, 29.9	0.99	99.7 ± 1.1a	27.8, 41.4	0.99
M2	98.4 ± 2.0a	21.1, 23.6	0.98	99.2 ± 1.5a	27.4, 41.7	0.99
M3	98.4 ± 2.1a	29.3, 23.6	0.98	99.2 ± 1.5a	41.1, 41.7	0.99
M4	100a	16.8, 19.2	1	100a	16.0, 17.6	1
M5	100a	16.8, 11.2	1	100a	16.0, 18.4	1
M6	100a	19.2, 11.2	1	100a	17.6, 18.4	1
1% w/w cypermethrin	100a	–	–	100a	–	–
70% v/v ethyl alcohol	0d	–	–	0d	–	–

Mean percentage knockdown rates in each column followed by a different letter are significantly different (one way ANOVA and Duncan’s multiple range test, *P* < 0.05).

*IMV (%) *Increasing Mortality Value, *EMI *Effective Mortality Index. Treatment codes are defined in Table 1.

Knockdown rates (K) at 60 min, mortality rates (M) at 24 h after exposure, KT_50_, effective knockdown index (EKI), and effective mortality index (EMI) of 0.25, 0.5 and 1.0% cinnamaldehyde against females of *Ae*. *aegypti* and *Ae*. *albopictus* are summarized in Table 5. At the highest concentration (1%), cinnamaldehyde showed the highest knockdown and mortality rates. All females of *Ae*. *albopictus* were more susceptible to cinnamaldehyde than *Ae*. *aegypti* females with a KT_50_ value ranging of 0.7 to 6.8 min (KT_90_ of 2.0–13.3 min) and 0.9 to 7.3 min (KT_90_ of 2.8–14.0 min), respectively. One percent cinnamaldehyde achieved the highest 100% knockdown and 100% mortality rates against both mosquito species and a KT_50_ of 0.7 to 0.9 min (KT_90_ of 2.0–2.8 min). These mortality and knockdown rates were equivalent to those provided by 1% w/v cypermethrin (which showed an EKI of 0.31 to 0.32 and an EMI of 1).

**Table 5:** Knockdown and mortality rates and KT_50_ of cinnamaldehyde against females of *Ae*. *aegypti* and *Ae*. *albopictus*.

Treatment	Species	Knockdown rate (%) ± SD at 60 min	Mortality rate (%) ± SD at 24 h	KT_50_ (min) (LCL-UCL)	KT_90_ (min) (LCL-UCL)	Slope ± SE	R^2^	Chi-square	EKI	EMI
	*Ae*. *aegypti*									
C1		100^ns^	100^ns^	7.3 (6.5–8.0)	14.0 (12.8–15.4)	0.192 ± 0.014	0.910	32.018	2.52	1
C2		100	100	5.0 (4.4–5.7)	10.7 (9.8–11.9)	0.224 ± 0.016	0.745	76.619	1.72	1
C3		100	100	0.9 (0.4–1.5)	2.8 (2.0–4.6)	0.675 ± 0.085	0.167	229.898	0.31	1
1% w/v cypermethrin		100	100	2.9 (2.6–3.2)	12.2 (8.5–14.3)	0.198 ± 0.015	0.763	108.531	–	
	*Ae*. *albopictus*									
C1		100	100	6.8 (6.1–7.5)	13.3 (12.2–14.7)	0.196 ± 0.014	0.896	36.512	3.09	1
C2		100	100	3.9 (3.4–4.5)	8.5 (7.7–9.5)	0.282 ± 0.022	0.808	54.382	1.77	1
C3		100	100	0.7 (–)	2.0 (–)	1.056 ± 0.127	0.063	58,184.288	0.32	1
1% w/v cypermethrin		100	100	2.2 (2.0–3.2)	7.4 (6.5–9.7)	0.232 ± 0.028	0.991	101.563	–	

*KT*_*50*_ 50% knockdown time, *R*^*2*^ regression coefficient, *LCL *lower confidence limit, *UCL *upper confidence limit, *EKI *Effective Knockdown Index, *EMI *Effective Mortality Index, *ns *not significant (*P* < 0.05). Treatment codes are defined in Table 1.

## Discussion

The essential oil yields from the barks of the three *Cinnamomum* species were in the range of 0.82–1.12% v/w. Several works reported a similar *C*. *verum* EO yield by steam distillation and hydro-distillation methods, such as 0.48% v/w^44^, 0.54% v/w^36^, and 1.14% v/w^49^. Some works also reported a similar *C*. *cassia* EO yield, for example, 0.72–2.38% v/w^49^, and 0.41–2.61% w/w^50–52^. The EO yields from several samples of *Cinnamomum* spp. showed some variations due to the different climates of different countries and different growth conditions (plant nutrition, soil fertilizer, and pest management, etc.) at the different cultivation sites. There are also other factors that influence yield such as harvesting time, growth stage of plant, age of bark, thickness of bark, density of oil cells in the bark^49^, and extraction method^50^. Moreover, extraction method also affects EO yield, and good cultivation management can increase EO yield^26,49,50^.

Although the EO yield of cinnamon is not high and cinnamon EOs are 10 times more expensive than common insecticides for mosquito control such as permethrin, cypermethrin, a cinnamon EO is much safer to humans and non-target organisms since it has been used as food ingredient for global populations since ancient times. The most important reason for using natural products from cinnamon, though, is that mosquito vectors have not developed resistance to them^20,21,25^.

Cinnamaldehyde was the major compound found from the three *Cinnamomum* spp. EOs. The cinnamaldehyde content ranged from 72.38 to 73.21% of the chemical composition. Several works reported similar cinnamaldehyde percentages in the chemical composition of *C*. *verum*, such as 64.66%^36^, 74.49%^49^, and 90.17%^44^. Other researchers reported that the cinnamaldehyde percentage in the composition of *C*. *cassia* EO was in the range of 68.52–76.40%^53,54^, and that the cinnamaldehyde percentage in *C*. *loureiroi* EO was 81.97%^50^. Cinnamaldehyde percentage in the composition of an extracted EO is a very important factor to consider because it is the main active constituent against mosquito vectors^53,54^; hence, the higher the better. Cinnamaldehyde has already been successfully used for mosquito control as well as several medicine and pharmacological applications^53,54^. Several factors that influence the percentage of cinnamaldehyde in a cinnamon EO were good agricultural management as well as good climate and environment^55^.

Moreover, the experimental conditions (temperature, relative humidity and photoperiod cycle) might affect the efficiency of EOs for mosquito control^56^. The temperature was 26 ± 2 °C, and the RH was 74 ± 4% RH with a photoperiod cycle of 12.5-h light: 11.5-h dark in this study. Under these conditions, the mortality rate exhibited by all *Cinnamomum* spp. EOs against females of *Ae*. *aegypti* and *Ae*. *albopictus* was in the range of 57.8–100%. These results agree well with a study by Soonwera and Sitthichock^26^. In that study, the post-application temperature of 25.3 ± 2.5 °C and an RH of 75.2 ± 3.4% of treatments of *C*. *citratus* and *E*. *globulus* EOs against *Ae*. *aegypti* and *Ae*. *albopictus* females yielded a mortality rate ranging from 59.2–100%. There have been reports that a high post-application temperature (30 °C) affected the efficacy of *Thymus vulgaris* EO against *Cx*. *quinquefasciatus* larvae: its LC_50_ was lower than that provided by a lower post-application temperature (15 °C)^56^. At the time that the experiments were designed, we did not consider that the actual usage temperature in Thailand and other tropical Asian countries might be a lot higher than our laboratory temperature, and so we did not devise an experiment to test the post-application temperature effect. In our future research, we would conduct experiments at an extreme temperature that might happen in Thailand and checked the EOs’ efficiency.

All combinations of *Cinnamomum* spp. EOs exhibited high, synergistic adulticidal activity against females of *Ae*. *aegypti* and *Ae*. *albopictus* with 100% mortality. Their increasing mortality value was improved from 11.2 to 41.1% compared to those of the individual EOs. One percent cinnamaldehyde showed the highest toxicity against both species with 100% knockdown and mortality rates and a KT_50_ and a KT_90_ ranging from 0.7–0.9 and 2.0–2.8 min, respectively. Although the toxicity of several EOs against adults of *Ae*. *aegypti* and *Ae*. *albopictus* were reported in previous studies, but the data on the efficacy of combinations of EOs from *Cinnamomum* spp. against females of *Ae*. *aegypti* and *Ae*. *albopictus* are limited. EOs of *C*. *verum* and *C*. *cassia* as well as cinnamaldehyde were previously found to be toxic against *Ae*. *aegypti* adult and larvae of *Ae*. *caspius* and *Cx*. *quinquefasciatus*^44,57,58^. Cinnamaldehyde showed toxicity against *Ae*. *aegypti* female adult with an LD_50_ of less than 3.5 µg/mg female^44^. *C*. *verum* EO and cinnamaldehyde also showed some toxicity to other insect pests (*M. domestica* and *Sitophilus oryzae*)^58,59^.

All EO combinations in these study showed a highly synergistic effect against females of the two mosquito species. EO combinations from *C*. *citratus* + *E*. *globulus* showed a highly synergistic effect against *Ae*. *aegypti* females with an improvement of more than 33% mortality rate increase^26^. Combined EOs from *C*. *cassia* + *Liex chinensis* inhibited growth and development of *Ae*. *caspius* larvae^57^. Combinations of cinnamaldehyde + limonene, cinnamaldehyde + carvacrol, and cinnamaldehyde + thymol showed a high toxicity and a synergistic effect against *Cx*. *quinquefasciatus*^42^. In contrast, a combination of *C*. *verum* EO + permethrin showed an antagonistic effect against *Ae*. *aegypti* females^36^.

The high toxicity and synergistic effect of all combinations of EOs in this study appear to be associated with their major composition, cinnamaldehyde. The mode of action of *Cinnamomum* spp. EOs against insect pests was permeability inhibition of cell membrane and disruption of intracellular enzymes^59,60^. Cinnamaldehyde inhibits the respiratory system of insects by inhibiting the enzymes involved in cytokinesis and reducing the ATPase activity of cell membrane, causing decreased cell respiration, decreased membrane depolarization, reduced membrane integrity and eventual mortality^58–60^.

More importantly, the combination of 2.5% *C*. *verum* + 2.5% *C*. *cassia* and individual 1% cinnamaldehyde were more toxic to both species of mosquitoes than cypermethrin. Cypermethrin is a neurotoxic chemical insecticide with a low LD_50_^10–13,26^ that affects the nervous, immune, and reproductive systems of humans^10,11,13^. Much safer than cypermethrin, *C*. *verum* and *C*. *cassia* EOs as well as cinnamaldehyde provided a high toxicity against mosquitoes but are non-toxic to humans, other mammals, or beneficial insects^19,20,58^. Furthermore, they are easily degraded in the environment, and they have already been used for ages by Asian people as an anti-microbial agent in their local medicine^45–48^.

To conclude, our objective of determining the insecticidal efficacies of *C*. *verum*, *C*. *cassia*, *C*. *loureiroi,* and their major constituents was fully achieved. According to the results, the combinations of EOs from *C*. *verum* + *C*. *cassia*, *C*. *cassia* + *C*. *loureiroi*, and *C*. *verum* + *C*. *loureiroi* showed a highly synergistic insecticidal effect against *Ae. aegypti* and *Ae*. *albopictus*. They have a high potential to be developed and improved into a spray formulation of eco-friendly adulticides for controlling or eradicating populations of *Ae*. *aegypti* and *Ae*. *albopictus* mosquitoes in urban and rural areas as well as for controlling dengue diseases and other vector-borne diseases. EOs from three *Cinnamomum* spp. barks may be the best source of alternative adulticides for sustainable mosquito control and safe for the environment and human health. Cinnamaldehyde, the major composition of the three *Cinnamomum* spp. also showed a high potential to be developed and improved into a new formulation of adulticides for controlling *Ae*. *aegypti* and *Ae*. *albopictus*. Some further research and development tasks are needed before the *Cinnamomum* spp. EO combinations and cinnamaldehyde can be used as adulticides in rural and urban areas. For example, their cost, safety, stability, post-application temperature effect, and other factors that may limit their use should be thoroughly investigated first.

## Methods

### Plant materials and essential oil extraction method

Dried barks of *C*. *verum*, *C*. *cassia*, and *C*. *loureiroi*, purchased from Chao Krompoe pharmacy, Chakkrawat, Bangkok 10100, Thailand*,* were extracted of their essential oils. Images of the three plant species and the chemical structure of their major constituent are shown in Fig. 1. Specimens of all *Cinnamomum* spp. were positively identified by a botanist from the botanical center, King Mongkut’s Institute of Technology Ladkrabang (KMITL), Bangkok, Thailand. All specimens were cleaned, crushed, and extracted of essential oils (EOs) by a hydro-distillation method^26–29^. After 6–7 h, the process was completed. Each EO was collected from the separating funnel, removed of water with anhydrous sodium sulfate (Na_2_SO_4_), preserved in airtight vials, and kept at 4 °C for further chemical composition analysis and bioassays (Table 1). All EOs and their combinations were diluted with ethyl alcohol into several formulations shown in Table 6.

**Table 6:** Formulations of individual cinnamaldehyde, individual *Cinnamomum* spp. essential oils, and their combinations in this study.

Code	Formulation
C1	0.25% cinnamaldehyde + 99.75% ethyl alcohol
C2	0.5% cinnamaldehyde + 99.50% ethyl alcohol
C3	1.0% cinnamaldehyde + 99.0% ethyl alcohol
CV1	1% *C. verum* EO + 99% ethyl alcohol
CC1	1% *C*. *cassia* EO + 99% ethyl alcohol
CL1	1% *C*. *loureiroi* EO + 99% ethyl alcohol
CV5	5% *C*. verum EO + 95% ethyl alcohol
CC5	5% *C*. *cassia* EO + 95% ethyl alcohol
CL5	5% *C*. *loureiroi* EO + 95% ethyl alcohol
M1	0.5% *C*. *verum* EO + 0.5% *C*. *cassia* EO + 99% ethyl alcohol
M2	0.5% *C*. *verum* EO + 0.5% *C*. *loureiroi* EO + 99% ethyl alcohol
M3	0.5% *C*. *cassia* EO + 0.5% *C*. *loureiroi* EO + 99% ethyl alcohol
M4	2.5% *C*. *verum* EO + 2.5% *C*. *cassia* EO + 95% ethyl alcohol
M5	2.5% *C*. *verum* EO + 2.5% *C*. *loureiroi* EO + 95% ethyl alcohol
M6	2.5% *C*. *cassia* EO + 2.5% *C*. *loureiroi* EO + 95% ethyl alcohol

### Chemical composition analysis of the three *Cinnamomum* spp. EOs

Chemical compositions of EOs from *C*. *verum*, *C*. *cassia*, and *C*. *loureiroi* were analyzed at the Center Laboratory of King Mongkut’s Institute of Technology Ladkrabang (KMITL), Bangkok, Thailand by Gas chromatography-mass spectrometry (GC–MS)^26^. The GC–MS analysis was performed with an Agilent Technology (USA) GC–MS system. All chemical constituents were identified with Agilent software (version G1701DA D.00.00) in combination with a mass spectral library from the National Institute of Standard and Technology (NIST; Wiley 7n.1). The GC–MS identified constituents were confirmed of their identity by comparing their retention indices to those of reference compounds reported in the literature. In this composition analysis, the RI of each chemical constituent was determined and calculated with respect to a homologous series of *n*-alkanes (C_7_–C_30_). Then, it was compared to the RI of a corresponding reference chemical reported in the literature^61,62^.

### Chemicals

Cinnamaldehyde, the major constituent of *C*. *verum*, *C*. *cassia*, and *C*. *loureiroi* EOs was purchased from Sigma-Aldrich Co., LTD., 3050 Spruce Street, Saint Louis, MO 63103, USA. The positive control was 1% w/v cypermethrin (Kumakai 10), manufactured by MD Industry Co. LTD., 22 Phahonyothin Rd., Wang-Noi district, Phranakhonsri Ayutthaya province, Thailand. The negative control was 70% v/v ethyl alcohol, manufactured by Hong Huat Co. LTD., 77/82-87 Krugthonburi Rd, Klongsarn, Bangkok 10600, Thailand.

### *Ae*.* aegypti *and *Ae*.* albopictus* rearing

Colonies of *Ae*. *aegypti* and *Ae*. *albopictus* were maintained at the entomological laboratory, Faculty of Agricultural Technology, KMITL, Bangkok, Thailand. The conditions in the laboratory were a temperature of 26 ± 2 °C and a 75 ± 5% RH with a photoperiod cycle of 12.5-h light: 11.5-h dark^26,31^. The eggs of *Ae*. *aegypti* and *Ae*. *albopictus* were obtained from the Mosquito Laboratory, KMITL. Eggs were hatched and reared for 1–2 days in a white plastic tray (the size of 23.0 cm wide × 32.0 cm long × 6.5 cm high) containing 2000 ml of clean water until the larvae emerged. A total of 200 larvae were reared in the white plastic tray and fed with fish food pellets one time per day for 12–14 days until they pupated. A total of 100 pupae were collected in a 250 ml beaker containing 200 ml of clean water and then transferred into an entomological cage (the size of 30 × 30 × 30 cm^3^). After 3–5 days, the pupae developed into adults that were reared in an entomological cage. Adults of both sexes were fed with 5% glucose solution + 5% multivitamin syrup solution. Two-day-old female adults of each mosquito species were used in an adulticidal bioassay^26,32^.

### Adulticidal bioassay

The toxicity of each EO, each formulation of combined EOs, and the major constituent of these EOs against female adults of *Ae*. *aegypti* and *Ae*. *albopictus* were determined by a standard WHO susceptibility assay^63^. A WHO susceptibility assay kit was purchased from the WHO Vector Control Unit in Penang, Malaysia. Following the WHO susceptibility assay guide lines^63^, 25 females of each mosquito species were exposed to 2 ml of each EO formulation (shown in Table 6). Namely, two millimeters of each formulation were dropped onto a filter paper (the size of 12 × 15 cm^2^) in the exposure tube (red spot tube, 4.4 cm in diameter and 12.5 cm in length). The mosquitoes were exposed to each formulation for 1 h and then transferred to the holding tube (green spot tube). The knockdown rate of each formulation against the two mosquito species was observed and recorded at 1, 5, 10, 30, and 60 min after exposure, while the mortality rate was observed and recorded at 24 h after exposure. The knockdown and mortality criterion were no movement of head, antenna, leg, wing, or other body parts^26,32^. Each treatment was performed in five replicates with positive (1% w/v cypermethrin) and negative (70% v/v ethyl alcohol) controls. The knockdown rate (K) and Mortality rate (M) were calculated by the following formula^26^.

Knockdown rate (%K) = [(K/T) × 100],

Mortality rate (%M) = [(M/T) × 100],

where K was the mean number of knocked-down adults; M was the mean number of dead adults; and T was the mean number of treated adults.

All tested *Ae*. *aegypti* and *Ae*. *albopictus* were kept under laboratory conditions post-application. The conditions were 26 ± 2 °C and 74 ± 4% RH with a photoperiod cycle of 12.5-h light: 11.5-h dark. All adulticidal bioassay was approved by the KMITL Ethic Committee, Ladkrabang, Bangkok, Thailand with a registration number, KDS 2018/001.

### Statistical analysis

The means and percentages of knockdown and mortality results were statistically analyzed by one-way analysis of variance (ANOVA). The means were compared by Duncan’s Multiple Range Test (DMRT) at *P* < 0.05. At the same *P* < 0.05, 50% Knockdown Time (KT_50_) was determined by a standard probit regression analysis (SPSS, Version 19)^26,29^.

- The increasing knockdown value (%IKV) was calculated by the following formula^26^:
  $$ \% {\text{IKV }} = \, \left[ {\left( {\% {\text{K of EOs combination }} - \, \% {\text{K of Individual EO}}} \right) \, / \, \% {\text{ K of EOs combination}}} \right] \, \times { 1}00 $$
- The increasing mortality value (%IMV) was calculated by the following formula^26^:
  $$ \% {\text{IMV }} = \, \left[ {\left( {\% {\text{M of EOs combination }} - \, \% {\text{M of Individual EO}}} \right) \, / \, \% {\text{ M of EOs combination}}} \right] \, \times { 1}00 $$
- The synergistic value (SV) of each formulation was calculated by the following
  formula^36^:
  $$ {\text{SV }} = \, \left[ {{\text{KT}}_{{{5}0}} {\text{of individual EO }}/{\text{ KT}}_{{{5}0}} {\text{of combined EOs}}} \right]. $$
  SV > 1 indicated that the combined EOs were synergistic; SV < 1 indicated that the combined EOs were antagonistic; and SV = 1 indicated that the combined EOs did not show any synergistic or antagonistic effect^36^.
- The effective knockdown index (EKI) was calculated by the following formula:
  $$ {\text{EKI }} = \, \left[ {{\text{KT}}_{{{5}0}} {\text{of individual EO or combined EOs }}/{\text{ KT}}_{{{5}0}} {\text{of 1}}\% {\text{ w}}/{\text{v cypermethrin}}} \right]. $$
  EKI < 1 indicated that the individual EO or combined EOs was more toxic than 1% w/v cypermethrin; EKI > 1 indicated that the individual EO or combined EOs was less toxic than 1% w/v cypermethrin; and EKI = 1 indicated that the individual EO or combined EOs was as toxic as 1% w/v cypermethrin.
- The effective mortality index (EMI) was calculated by the following formula:
  $$ {\text{EMI }} = \, \left[ {\% {\text{M of individual EO or combined EOs }}/ \, \% {\text{M of 1}}\% {\text{ w}}/{\text{v cypermethrin}}} \right]. $$

EMI = 0 or > 1 indicated that the individual EO or combined EOs was more toxic than 1%w/v cypermethrin, and EMI < 1 indicated that the individual EO or combined EOs was less toxic than 1% w/v cypermethrin.
